# An advanced vision of magnetocardiography as an unrivalled method for a more comprehensive non-invasive clinical electrophysiological assessment^☆^

**DOI:** 10.1016/j.ahjo.2025.100514

**Published:** 2025-02-23

**Authors:** Riccardo Fenici, Marco Picerni, Peter Fenici, Donatella Brisinda

**Affiliations:** a Biomagnetism and Clinical Physiology International Center, Rome, Italy; b International School for Advanced Studies (SISSA); c Catholic University of the Sacred Heart, School of Medicine and Surgery; d Fondazione Policlinico Universitario Agostino Gemelli, IRCCS, Rome, Italy

**Keywords:** Magnetocardiography, Non-invasive multimodal imaging of electrophysiologic events, Threedimensional localization of arrhythmogenic substrates, Ischemic injury current, Mathematical modeling, Action potential, Electrotonic current

## Abstract

Decades of experimental and clinical studies, along with the most recent clinical trials, have demonstrated the diagnostic potential of magnetocardiography, particularly for the non-invasive early diagnosis of myocardial ischemia. It has also proven to be a valuable clinical tool for monitoring fetal well-being, normal growth, prenatal arrhythmias, and risk markers for sudden death. Such applications have recently received official recognition from Health Canada and the American Heart Association. This unquestionable success, and the additional evidence of magnetocardiography's high sensitivity in diagnosing infiltrative and inflammatory cardiomyopathies, has sparked renewed interest among clinicians.

However, while these aforementioned applications are likely to significantly influence the broader clinical adoption of magnetocardiography, the general focus on these areas has shifted attention away from what we have always regarded as the fundamental strength of contactless cardiac magnetic field mapping: its unique ability to bridge the gap between experimental electrophysiology at the cellular level and non-invasive clinical assessments of human electrophysiology.

This review aims to engage readers by sharing our vision, experience, and several key research milestones, emphasizing the lesser-explored yet significant potential of magnetocardiography. Specifically, it highlights its unique capability to detect electrically silent phenomena that may be critical for the timely and accurate identification of arrhythmogenic focal electrotonic and vortex currents, which can trigger or sustain life-threatening arrhythmias.

## 1 Introduction

More than 60 years after the first recording of the heart's magnetic field [1], magnetocardiography (MCG) is now at a critical turning point. The results of numerous experimental and clinical studies [2,3], particularly the recent MAGNETO [4] and MICRO [5] clinical trials have demonstrated its significant diagnostic potential, especially for the early non-invasive diagnosis of myocardial ischemia. In recognition of this, Health Canada has granted the CardioFlux Magnetocardiograph a license for clinical use to aid in diagnosing myocardial ischemia [6]. This undeniable success supports decades of global research dedicated to demonstrating the clinical utility of contactless MCG, particularly its ability to enhance diagnostic accuracy for myocardial ischemia without the need for contrast agents, radiation, or stress-inducing drugs.

Clinical interest in fetal MCG (FMCG) has also grown significantly over the past decade. It is now recognized as a valuable clinical tool for monitoring fetal well-being, assessing normal growth, and identifying prenatal arrhythmias and risk markers for sudden death [[7], [8], [9], [10]]. Recent reviews have thoroughly summarized the related literature [2,3,11,12]. Additionally, several clinical studies have reported good diagnostic sensitivity for non-ischemic, infiltrative, and inflammatory cardiomyopathies [[13], [14], [15], [16], [17], [18], [19], [20]], further sparking renewed interest in MCG. This progress has been driven by advances in non-cryogenic magnetic sensor technology and significant research and development investments aimed at creating more compact, portable, and even wearable MCG devices [21,22].

However, while the above-mentioned applications will significantly impact the broader clinical adoption of MCG, the general focus on these areas has shifted attention away from what we have always regarded as the fundamental strength of MCG: its unique ability to bridge the gap between experimental electrophysiology at the cellular level and comprehensive, non-invasive clinical assessments of human electrophysiology.

In alignment with this original vision, we have dedicated decades of research since the early 1980s to establish a clinical setup where this concept could be explored and validated whenever possible [23]. Encouraged by our ongoing efforts, many other researchers have conducted significant clinical studies demonstrating MCG's superior accuracy compared to electrocardiography in localizing arrhythmogenic substrates and assessing arrhythmogenic risk non-invasively [[24], [25], [26], [27], [28], [29], [30], [31], [32], [33], [34], [35]]. The clinical relevance of these findings has been comprehensively summarized in review articles [21,[36], [37], [38], [39], [40]], yet it remains largely underappreciated in clinical practice.

Therefore, this review aims to engage readers by sharing our vision, experience, and several key research milestones, providing clear evidence that clinical recording of the cardiac magnetic field could be a sensitive, contactless method for studying human electrophysiological phenomena at a quasi-cellular level. This approach may ultimately bridge the gap between experimental cellular and non-invasive clinical electrophysiology.

## 2 Milestones in the magnetic study of electrophysiology

### 2.1 The first direct-current (DC) MCG

Since the introduction of MCG in humans, there has been an ongoing debate about whether the technically demanding recording of cardiac magnetic fields can provide additional electrophysiological insights relevant to clinical practice, compared to the less expensive and more standardized methods of electrocardiology. While theoretical physics and mathematical modeling have generally supported this idea (though not unanimously) [[41], [42], [43], [44], [45]], fundamental experimental studies also offer compelling evidence.

The first groundbreaking experimental demonstration of MCG's ability to detect cardiac electrophysiological events that are undetectable with ECG was published by David Cohen in 1971 [46]. He used direct-current (DC) SQUID magnetic sensors to develop the DC-MCG. This method was employed to non-invasively investigate the electrophysiological mechanisms underlying the so-called “current of injury,” which generates the upward S-T shift on the ECG following experimental myocardial infarction in dogs [46].

As well known since the 1950s [47], ischemic injury leads to incomplete repolarization of ischemic cardiomyocytes, which remain partially depolarized. The resulting potential gradient between the ischemic and surrounding healthy myocardium generates a current that flows from the injured tissue toward the healthy tissue, producing a nonzero magnetic field during diastole. During the S-T interval, however, the healthy myocardium also becomes depolarized, nullifying the injury current. This “injury current” cannot be detected by standard ECG, which requires high-pass filtering (usually at least 0.05 Hz) to avoid artifacts from electrode polarization and the galvanic skin effect. However, it was detected using DC-MCG in the MIT magnetically shielded room, which provided a stable internal magnetic field suitable for accurately detecting changes in the magnetic DC field during experimental coronary occlusion in dogs.

While the results of MIT's initial pilot study were inconclusive in clarifying the relationship between the S-T shift and the ischemia-induced injury current, a subsequent DC-MCG study, conducted with more rigorous methodology in intact-chest dogs, definitively demonstrated that during acute ischemia caused by coronary occlusion, the S-T shift recorded with surface ECG is a secondary effect of a primary steady injury current, which is interrupted during the S-T interval [48].

Interestingly, while DC-MCG is technically demanding and may not be practical for routine clinical use, it has played an important role in clarifying the mechanism of S-T depression induced by exercise stress testing in patients with typical coronary artery disease. Consistent with the experimental findings, preliminary human DC-MCG recordings showed that exercise-induced S-T depression in a patient with hemodynamically significant coronary artery occlusion is primarily caused by an injury current, which is interrupted during the S-T interval [49]. In contrast, S-T shifts observed in patients with the ECG early repolarization pattern or with left bundle branch block are caused by a current that flows only during systole, presumably due to altered ventricular repolarization [50].

### 2.2 First magnetic field studies at the cellular and tissue levels

Since the 1980s, John Wikswo and his team have developed several innovative experimental approaches that highlight the powerful capability of biomagnetic measurements to non-invasively provide fundamental electrophysiological information at both the cellular and tissue levels [51].

The original recording of the magnetic field from the frog sciatic nerve using a mini toroid magnetic sensor provided the first direct estimate of the relationship between the nerve's transmembrane action potential, the induced current, and the corresponding magnetic field amplitude [52]. This impressive experimental model demonstrated the strong potential of contactless biomagnetic recordings to obtain functional electrophysiological information from outside the cell membrane of intact excitable cells. While this innovative technique was quickly translated into clinical practice [53], more advanced methods for in vitro biomagnetic imaging of the magnetic field produced by cardiac muscle were subsequently refined [[54], [55], [56]].

Further progress included the invention of the ‘SQUID microscope,’ which enabled high-resolution magnetocardiographic mapping on the surface of an isolated Langendorff-perfused rabbit heart. This system was optimized with a 500 μm diameter pickup coil and a field sensitivity of 330 fT Hz^−½^ for frequencies above 1 Hz, allowing for the imaging of magnetic fields associated with current injection and the propagation of action currents in cardiac tissue [57]. In addition, a mathematical technique based on Fourier transforms was developed to determine the two-dimensional current distribution from magnetic field measurements by solving the inverse problem restricted to a two-dimensional plane [58].

With a more advanced experimental setup, Holtzer et al. combined high-resolution magnetic imaging of the action magnetic field generated by planar activation wavefronts on the left ventricular wall of Langendorff-perfused isolated rabbit hearts with optical imaging of the transmembrane potentials [59].

Using the same experimental setup, McBride et al. [60] demonstrated that the pacing-induced electrical activation of the apex's spiral fibers in the isolated rabbit heart generates not only the radial currents detectable through optical mapping of transmembrane action potentials but also electrically silent vortex currents, which were only detected by recording the Bz magnetic field component. They observed a difference between the simple circular wavefront reconstructed from the transmembrane potentials and the more complex spatial activation pattern inferred from the magnetic recordings and concluded that the magnetic data contained information that could not be obtained through electrical measurements alone.

### 2.3 Modeling of transmembrane action potential and related magnetic field

The relationship between transmembrane action potentials and their associated magnetic fields has been studied in silico through computational modeling. In 2008, a computational study investigated reentry wavefront propagation induced by the S1–S2 pacing protocol, using mathematical models of human cardiac cells to simulate atrial and ventricular action potentials and the corresponding magnetic field distribution [61]. The authors calculated the magnetic field patterns generated by the propagation of atrial and ventricular reentry wavefronts, comparing the magnitudes of normal action potentials with those measured during reentry propagation. They also analyzed temporal changes in the magnetic field maps, focusing on the direction, strength, and vector angle shifts of the MCG-based current dipole as the reentry wavefronts moved in a rotating spiral pattern. This analysis demonstrated that MCG patterns are valuable for the theoretical study of reentry waves.

A more recent modeling study by Crispino et al. [62], aimed to bridge the gap between electrophysiological indicators and magnetic field measurements at the cellular scale. In this study, cardiac tissue was modeled as a straight excitable wire. Action potentials were simulated using a simplified (four-variable) model, and the effects of temperature on action potential duration (measured at 80 % repolarization), conduction velocity, and cycle length-related restitution curves were calculated. The corresponding magnetic field was determined using the Bio-Savart law. The study also evaluated cardiac alternans by comparing restitution curves derived from action potential duration with those based on the magnetic field generated by simulated cardiac electrical activity at various temperatures.

The study confirmed a strong relationship between the features of the action potential and the corresponding temporal and spatial behavior of the magnetic field. Notably, it provided previously unreported evidence that magnetic field-based detection of cardiac alternans is more efficient and “*could substitute for, or even outperform*,” detection based on electrical parameters.

The authors concluded that the strong correlation between the magnetic field and cellular electrical activity opens up exciting possibilities for non-invasive investigation of cardiac electrophysiology, enabling the study of biological samples in physiological conditions without direct cell access.

### 2.4 Filling the gap between experimental and clinical magnetic electrophysiology

Overall, the experimental demonstration that biomagnetic recordings enable contactless investigation, imaging, localization, and quantitative assessment of primary electrophysiological sources (i.e., cardiac impressed currents) supports our original vision that, if these capabilities are transferable to clinical practice, MCG could become a revolutionary method for comprehensive, contactless assessment of human cardiac electrophysiology [37,63,64].

**Fig. 1:**
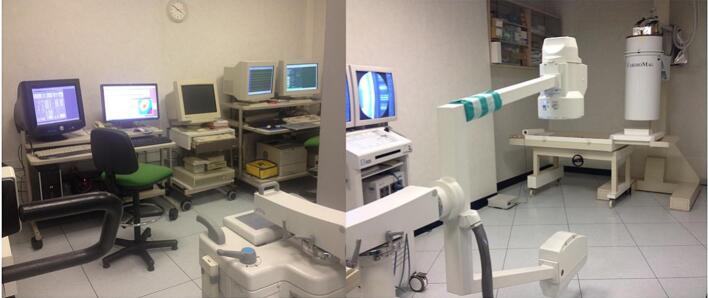
Overview of the amagnetic cardiac catheterization laboratory at the Catholic University.

However, forty years ago, this vision was often dismissed as unreliable—if not outright “crazy”. To prove and validate the concept, we had to invent and build the unique “amagnetic cardiac catheterization laboratory” (Fig. 1) and the “*Biomagnetically localizable multipurpose catheter and method for magnetocardiographic guided intracardiac mapping….*” (patented with the Italian CNR) [65], to enable MCG mapping even during invasive electrophysiological procedures clinically indicated for diagnostic and/or interventional purposes. The amagnetic catheter enabled MCG-guided single-catheter percutaneous endocardial recording of monophasic action potentials (MAP) and the intracardiac injection of artificial dipolar current sources of varying geometry and strength. Despite its obvious limitations, this innovative approach sought to replicate, during clinical electrophysiological studies, an experimental setup similar to that developed by Wikswo et al. [52]. The three-dimensional (3D) localization accuracy of the distal terminal of an amagnetic catheter prototype, using unshielded MCG mapping and the equivalent current dipole inverse solution in comparisons with its fluoroscopic position, was reported since the 1980s [66,67]. The first international consensus on the MCG localization accuracy of intracardiac sources [68,69] and on the clinical relevance of non-invasive biomagnetic imaging for the ablation of cardiac arrhythmias was reached at a workshop held in Rome in 1989, on behalf of the “Concerted Action on Biomagnetism” and supported by the Medical and Health Research Program of the European Communities [36]. This occurred notably well before the advent of the first system for interventional electroanatomical imaging [70].

**Fig. 2:**
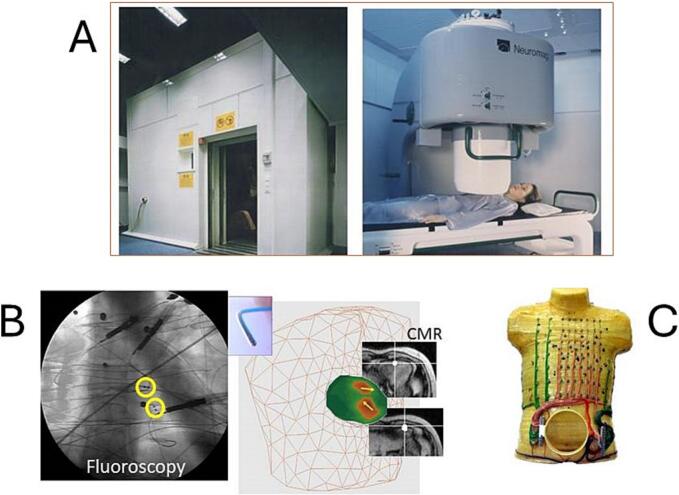
(A) The Helsinki BioMag Center Neuromag MCG system. (B) Example of MCG current density imaging (yellow arrows on the green heart model, and white dots in CMR images) of two amagnetic catheters positioned at the antero-septal and apical wall of the right ventricle (highlighted with yellow circles on the frontal fluoroscopy). (C) The realistic torso phantom for simultaneous MCG and Body surface potential mapping experiments. (For interpretation of the references to colour in this figure legend, the reader is referred to the web version of this article.)

Further validation of the “amagnetic catheter technique” was conducted using the state-of-the-art Neuromag multichannel system in the shielded room of the Helsinki University Central Hospital (HUCH) BioMag Center, as part of the European Communities' Birch Programme. In patients, the mean 3D mismatch between fluoroscopic and MCG localization of the AC was 6 ± 2 mm (based on beat-to-beat analysis), with a coefficient of variation of 1.37 % and a coefficient of reproducibility of 2.6 mm [[71], [72], [73], [74]] (Fig. 2). In the realistic torso phantom, the 3D localization accuracy of the amagnetic catheter's dipoles was 2 ± 1 mm, which was twice as accurate as the results obtained using the simultaneous inverse solution from 138-electrode body surface potential mapping [75]. Interestingly, a similar superiority in magnetic localization accuracy was later confirmed by a phantom study that assessed the localization reproducibility and spatial accuracy of a sensor-based electromagnetic navigation system for interventional electrophysiology [76].

**Fig. 3:**
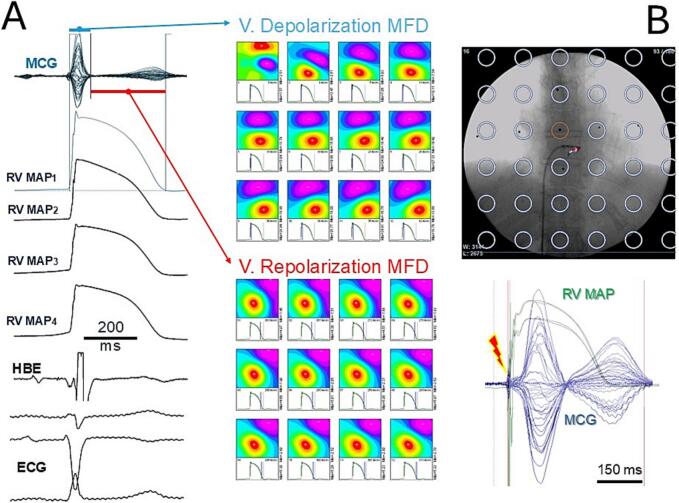
(A) Example of simultaneous MCG mapping and single-catheter right ventricular MultiMap recording during sinus rhythm. In (B), the overlay of contactless MCG imaging of the distal terminal of the amagnetic catheter on its fluoroscopic imaging during the EP study. All MCG traces are shown in the “butterfly” mode. MFD: Magnetic Field Distribution. HBE: His Bundle Electrogram. V: ventricular.

Improved versions of the amagnetic catheter [77,78], have been developed for MCG-compatible single-catheter percutaneous endocardial mapping of multiple monophasic action potentials (MultiMAP) (Fig. 3A), as well as for non-fluoroscopic imaging of the amagnetic catheter's distal end (Fig. 3B).

These innovations offer a novel method for non-fluoroscopic magnetic navigation of the amagnetic catheter and enable minimally invasive, single-catheter high-resolution electrophysiological studies of arrhythmogenic substrates pre-interventionally localized [79] through multimodal integration of MCG source imaging (MSI) with MRI or CT anatomical images [80,81].

**Fig. 4:**
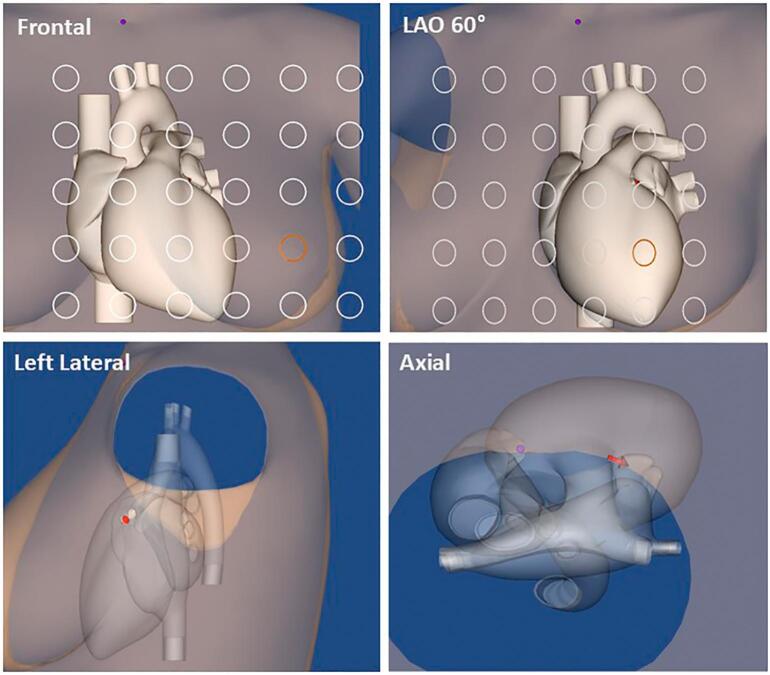
Four standard projections of the 3D elastic heart model scalable to the patient's sizes. By changing the degree of transparency is possible to visualize the localization of the source of interest (red dipole arrow). (For interpretation of the references to colour in this figure legend, the reader is referred to the web version of this article.)

**Fig. 5:**
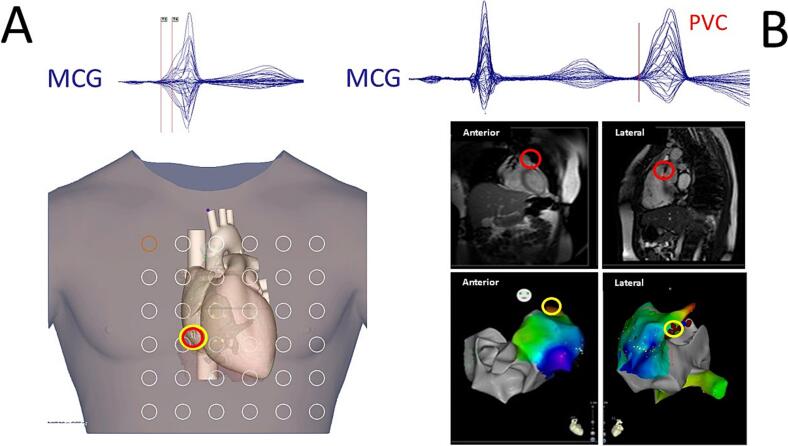
Examples of successfully ablated MCG localized arrhythmogenic substrates: (A) Right lateral accessory pathway (multimodal MCG-CT-Carto integration). (B) Right ventricular outflow tract (RVOT) bigeminy (multimodal MCG-MRI-Carto integration). The sites of MCG localization and successful ablation are highlighted with red and yellow circles respectively. MCG: “Butterfly” overlays of the 36-channel waveforms). (For interpretation of the references to colour in this figure legend, the reader is referred to the web version of this article.)

To streamline this multimodal integration, the MCG localization results were automatically visualized within an “elastic” 3D MRI-based model of the heart, tailored to the individual patient's size (Fig. 4, Fig. 5), refined using software that generates a realistic 3D heart from patient's 2D orthogonal fluoroscopic images [82].

In the Rome unshielded biomagnetism catheterization room, the localization precision of the improved amagnetic catheter dipoles in a simplified phantom was 1.16 ± 0.42 mm, demonstrating optimal repeatability (coefficient of variation ± standard error: 0.79 ± 0.43 %), with a 3D absolute error of 0.26 ± 0.25 cm [83]. In patients, the average 3D localization accuracy of the amagnetic catheter, validated by comparison with its fluoroscopic position, was better than 8 mm. This level of accuracy was more than sufficient for the preintervention localization of arrhythmogenic substrates, which were successfully treated with catheter ablation. Examples of the multimodal integration of MCG source imaging (MSI) with CT scan and MRI are shown in Fig. 5A and B, respectively [80,81].

**Fig. 6:**
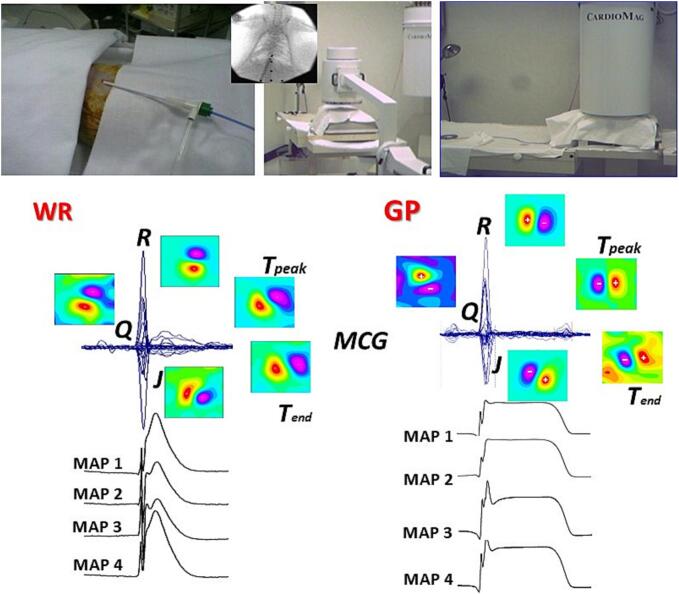
Experimental set-up for percutaneous ventricular MultiMap recording and simultaneous MCG mapping in the spontaneously breathing intact small rodents. WR: Wistar Rat; GP guinea pig.

The high-resolution performance of simultaneous MultiMap recordings and unshielded MCG mapping was also experimentally tested on intact rodents [[84], [85], [86]] (Fig. 6).

**Fig. 7:**
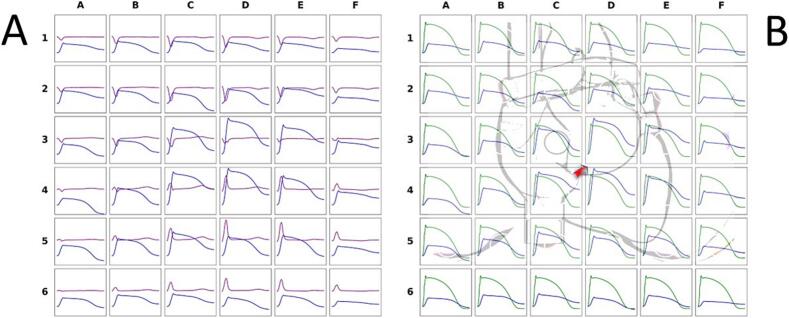
(A) Action potential (blue waveform) calculated at each MCG grid point from MCG mapping (violet waveform). (B) Direct comparison between the action potential waveform (blue) and the right ventricular monophasic action potential (green) recorded with the MCG-localized amagnetic catheter at the site marked by a red arrow (shown by the scaled overlay of the 3D heart model on the MCG mapping grid) (same patient as in Fig. 3). (For interpretation of the references to colour in this figure legend, the reader is referred to the web version of this article.)

In 2004, our vision that contactless MCG mapping could significantly enhance the noninvasive clinical evaluation of cardiac electrophysiology, almost at the tissue scale, was supported by a study [87] that described a mathematical method for the noninvasive reconstruction of the ventricular action potential waveform using magnetocardiographic signals from a patient with congenital type-1 Long-QT syndrome. Briefly, the noninvasive reconstruction of the ventricular action potential waveform was derived by calculating the current-arrow map (CAM), which was obtained from the derivatives of the cardiac magnetic field component normal to the anterior chest plane (Bz), as outlined by Hosaka and Cohen [88]. In the reported Long-QT-1 patient, the MCG-based reconstruction of the AP waveform enabled the non-invasive detection and localization (in the right ventricle) of a potentially arrhythmogenic phenomenon (EAD-triggered activity) validated by comparison with that of a separately recorded right ventricular monophasic action potential (RVMAP) [87]. We have compared the AP waveforms calculated from MCG mapping with those of the RVMAP, simultaneously recorded with the amagnetic catheter. The morphology of the MCG-calculated AP was in good agreement with that of the RVMAP in about half of the 36 MCG recording positions. However, the AP calculated from several MCG grid points did not return to the baseline at the end of repolarization (Fig. 7).

Finally, another intriguing piece of evidence comes from a very recent clinical MCG study that proposes a novel method, called Magnetoionography (MIG), derived from MCG mapping. This method aims to non-invasively characterize individual ionic transmembrane and intracellular currents of cardiomyocytes [89]. The rationale put forward by the authors is that, since the membranes of cardiac cells are completely permeable to the intracellularly generated magnetic fields, it is possible to indirectly calculate parameters that provide a quantitative estimate of the transmembrane and intracellular ionic currents by applying novel algorithms for MCG data processing [90].

## 3 Discussion

This review highlights the lesser-explored yet significant potential of MCG in the non-invasive clinical detection of arrhythmogenic mechanisms. While MCG's established role in localizing intracardiac sources [[72], [73], [74],^83^], and facilitating preintervention planning [81,[91], [92], [93], [94], [95], [96], [97], [98]] and arrhythmogenic risk assessment [40], is well-documented, this review emphasizes its additional capability in detecting electrically silent phenomena [46,49,87,99] that may be critical for a mechanistic understanding of arrhythmogenic focal triggers and vortex currents that may induce sustained complex arrhythmias [54,[60], [61], [62],^100^]. However, there are still problems to be solved.

The experimental demonstration of DC-MCG's ability to clinically measure the “injury current” during acute ischemia induced by coronary artery ligation in a canine model represents a significant milestone in MCG development [46]. This work not only clarified the diastolic nature of the injury current but also paved the way for further pioneering research. Notably, MCG was later shown to detect a diastolic DC-current in a patient with effort-induced ischemia [49], marking the first human evidence that DC-MCG can differentiate between “true” ECG ST shifts, which are likely caused by systolic currents [50], and the ischemia-induced steady diastolic potential gradient injury current resulting from depressed repolarization in ischemic myocardium. This diastolic potential gradient, when amplified, can lead to electrotonic currents and become arrhythmogenic [101]. With the anticipated advancements in the sensitivity and spatial resolution of new biomagnetic sensors, MCG current density imaging holds considerable promise in detecting electrotonic currents before they escalate into premature ectopic beats, which could trigger sustained, life-threatening arrhythmias. A major obstacle to the widespread clinical application of DC-MCG is the interference from noncardiac DC-currents, particularly those originating from gastrointestinal sources highlighting a significant challenge for routine clinical use and suggesting the need for further refinement in sensor technology and denoising techniques [49].

While it is theoretically possible to calculate the cardiac AP from MCG signals, directly reconstructing the full transmembrane AP waveform remains highly challenging due to the indirect relationship between the magnetic field and cellular electrical activity. The complexity arises because the action potential is a local event that occurs at the level of individual cardiomyocytes, whereas the magnetic field measured by MCG is the result of the simultaneous electrical activity of multiple cardiomyocytes and is proportional to the current density generated by an undefined volume of underlying cardiac tissue. This averaging effect complicates the ability to directly correlate the magnetic signal to the precise transmembrane potential of individual cells.

Despite these challenges, the first tentative attempts to reconstruct the cardiac action potential waveform from contactless MCG mapping have yielded fascinating preliminary results. The preliminary findings that the reconstructed AP waveform from MCG data can align with the waveform of the local MAP invasively recorded in close anatomical proximity offer promising evidence for the potential of MCG to provide novel non-invasive insights into cardiac electrophysiology [87]. In our experience, comparing the AP waveform calculated from MCG with that of the simultaneously recorded RVMAP (Fig. 7) confirms the feasibility of the method. However, some incongruences remain, such as the lack of baseline recovery following the end of repolarization, which requires further investigation. These discrepancies suggest that while MCG-derived AP waveforms show promising potential, more work is needed to refine the approach.

To address these issues, additional research is warranted, particularly through the use of advanced computational modeling [61,62,102]. Such modeling can help to simulate how local action potentials from different regions of the heart contribute to the magnetic fields measured by MCG. Moreover, determining the optimal sensor density for MCG that allows for accurate calculation of local APs—sufficient for diagnostic purposes—will be crucial for improving the precision and clinical applicability of the method. Advanced signal processing techniques, including deep learning algorithms [103] and software tools, could enhance the correlation of MCG signals with known AP waveforms. Additionally, their multimodal integration with anatomical imaging [97,104] could improve the reconstruction of AP from measured magnetic fields, offering better spatial accuracy.

Finally, further validation of the MCG's potential to bridge the gap between cellular and clinical electrophysiology is provided by research from the Catholic University using the amagnetic catheter technique [80], as well as recent publications on the preliminary clinical application of the novel Magnetoionography (MIG) method developed in Germany [89,90].

**Fig. 8:**
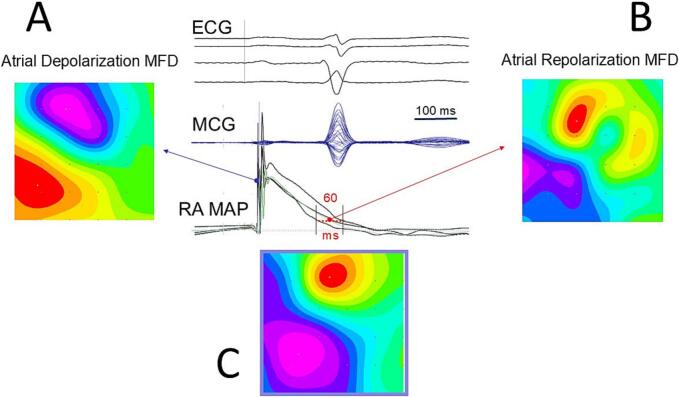
Patient with paroxysmal atrial fibrillation. (A) Normal magnetic field distribution (MFD) during atrial depolarization. (B) Abnormal fragmentation of magnetic field distribution during atrial repolarization (for comparison, the atrial repolarization magnetic field distribution of a normal subject is shown in (C). Simultaneous right atrial MultiMap recording evidences abnormal (60 ms) dispersion of right atrial action potentials' duration.

**Fig. 9:**
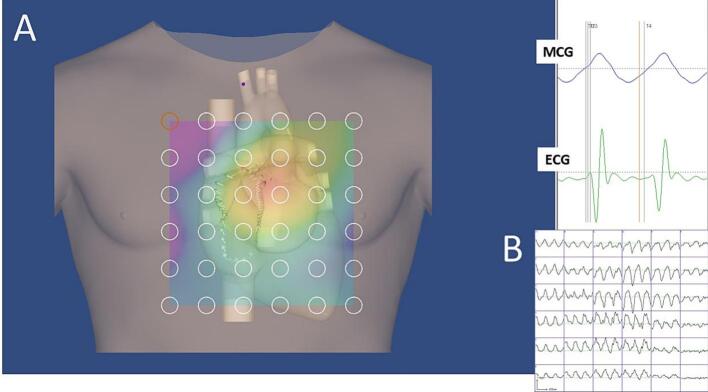
(A) MCG imaging of a right-sided clockwise macro-reentry (sequence of the magnetic dipoles localization) in a patient with type-2 atrial flutter. (B) 36-channel MCG of the flutter waves. The white circles indicate the MCG sensors' position.

A notable example from the former institution highlights the high sensitivity of MCG imaging in detecting arrhythmogenic atrial repolarization dyshomogeneity in a patient with paroxysmal atrial fibrillation, validated by simultaneous atrial MultiMap recordings that showed local dispersion in the duration of right atrial action potentials (Fig. 8). Additionally, in another patient (Fig. 9), 3D MCG imaging captured a right-sided reentry circuit during spontaneous type-2 atrial flutter, confirming the clinical feasibility of MCG imaging of atrial reentry wave propagation, as theoretically simulated by Im et al. in a three-dimensional human heart model [61].

The study by Wessel et al. [89] evaluated the ability of MCG to predict sudden death in patients with acute chest pain. This study provided the first clinical demonstration showing that three of the nine calculated MIG parameters, which are sensitive to the dynamics of cellular ion currents, were significantly different between patients admitted to the hospital with chest pain without ST-segment elevation who survived and those who did not, over a 6.5-year follow-up period. A recent editorial by Park et al. [90] explains how MIG enables the non-invasive measurement of magnetic parameters sensitive to transmembrane calcium and potassium ion currents, as well as subcellular Ca^2+^ transients. The reported differences between the MIG patterns of a young, healthy male and a 76-year-old healthy male, as well as between patients with diabetes, heart failure with reduced ejection fraction, and those with post-COVID disease, are promising. If these preliminary findings are validated through further targeted studies, MIG could become an innovative method for non-contact clinical assessment of human electrophysiology at the quasi-cellular level, with potential applications in monitoring the effects of cardioactive drugs. Interestingly, a previous experimental study investigating the effects of extremely low-frequency and weak magnetic fields (LF-WMF) on ionic transients, particularly Ca^2+^, may indirectly support the rationale behind Magnetoionography, confirming the link between magnetic fields and cardiomyocyte Ca^2+^ transients [105].

## 4 Conclusions

Magnetocardiography (MCG) took over 60 years to receive official approval as a medical device. This milestone was reached recently when Health Canada authorized the CardioFlux magnetocardiograph for diagnosing myocardial ischemia. Despite this achievement, also supported by multiple recent studies [2,3,[106], [107], [108], [109], [110]], we still lack a full mechanistic understanding of why just 90 s of radiation-free rest-MCG can deliver predictive values that rival those of effort ECG testing and are nearly equivalent to the results provided by nuclear medicine.

A potential explanation can be found in this (perhaps provocative) review of some pioneering experimental and clinical research, which we have integrated into our original vision. This vision is grounded in a belief in the laws of physics and theoretical suggestions that biomagnetic fields contain additional information beyond conventional electrocardiological methods. Indeed, the direct connection between the cardiac magnetic field and the primary electrophysiological sources (the cardiac currents), the minimal distortion of the magnetic field as it passes through tissues with different conductivities, and the simpler solution to the magnetic inverse problem are all factors that favor the “wireless” biomagnetic diagnostic approach. Furthermore, the ability to reconstruct the action potential waveform and assess ion currents using clinical MCG—an achievement once possible only in experimental settings—strongly supports these theoretical claims.

As more advanced processing tools are developed to exploit the unique advantages of MCG, we are likely to gain a mechanistic understanding of its higher sensitivity in diagnosing myocardial ischemia in clinical settings. However, we believe this is just one of the many compelling features of MCG. Much more will be uncovered as advances in sensor technology and denoising techniques make MCG instrumentation more affordable and easier to use at scale. This will enable reliable operation in unshielded hospital environments, facilitating wider clinical adoption and enabling a shared diagnostic experience with standardized protocols for data acquisition and analysis [21].

## CRediT authorship contribution statement

**Riccardo Fenici:** Conceptualization, Formal analysis, Investigation, Project administration, Supervision, Writing – review & editing, Funding acquisition, Writing – original draft. **Marco Picerni:** Formal analysis, Software, Data curation, Writing – review & editing. **Peter Fenici:** Writing – review & editing. **Donatella Brisinda:** Conceptualization, Data curation, Formal analysis, Investigation, Validation, Writing – review & editing.

## Declaration of competing interest

The authors declare that they have no known competing financial interests or personal relationships that could have appeared to influence the work reported in this paper.
